# Pattern of presentation of Graves’ disease and response to radioiodine therapy in South African men

**DOI:** 10.11604/pamj.2018.29.48.13655

**Published:** 2018-01-18

**Authors:** Yetunde Ajoke Onimode, David Magbagbeola Dairo, Annare Ellmann

**Affiliations:** 1 Nuclear Medicine Unit, Department of Radiation Oncology, College of Medicine, Faculty of Clinical Sciences, University of Ibadan, Oyo State, Nigeria; 2 Nuclear Medicine Centre, University College Hospital, Ibadan, Oyo State, Nigeria; 3 Department of Epidemiology & Medical Statistics, College of Medicine, Faculty of Public Health, University of Ibadan, Oyo State, Nigeria; 4 Department of Epidemiology & Medical Statistics, University College Hospital, Ibadan, Oyo State, Nigeria; 5 Division of Nuclear Medicine, Department of Medical Imaging & Clinical Oncology, Tygerberg Hospital, Western Cape Province, South Africa; 6 Faculty of Medicine and Health Sciences, Stellenbosch University, Western Cape Province, South Africa

**Keywords:** Hyperthyroidism, thyroid neoplasms, Graves´ disease, nuclear medicine

## Abstract

**Introduction:**

Typically hyperthyroidism has been more often associated with the female gender. There is a large female predilection (male:female sex ratio up to 1:10), with little documentation in the literature about wholly male hyperthyroid populations. A male incidence of 0.7 per 100, 000 has been reported for South African men while the women have a relatively higher rate of 0.02. There is no documented evidence between male and female genders in response to treatment of PH with radioactive iodine (RAI), although operational evidence suggests that hyperthyroidism in males is less amenable to RAI treatment (RAIT) than females. This study therefore proposed to evaluate male hyperthyroid patients with Graves' disease (GD) treated at our facility, for factors affecting outcome of RAIT.

**Methods:**

This is a retrospective analysis of records of hyperthyroid patients who were treated with RAI over a 19-year period at a university teaching hospital, in the Western Cape of South Africa.

**Results:**

The overall cure rate was 76.4% for these male patients. Cure was observed as euthyroidism in 31 patients (15.3%) and hypothyroidism in 129 (63.5%). Age, thyroid uptake, severity of hyperthyroidism, previous antithyroid drug (ATD) usage, administered quantity of RAI, ethnicity and patients' pulse at presentation were not significant in influencing outcome.

**Conclusion:**

Factors which have been evaluated as affecting outcome of RAIT were unimportant in these patients. Despite the mainly hyperthyroid presentation of the patients, RAIT was so effective that the main type of cure after therapy was hypothyroidism.

## Introduction

Graves' disease (GD) was named after Robert Graves for his publication in 1835 on the features of tachycardia and goitre in a trio of women, along with exophthalmos in a fourth [1]. However, GD was reportedly first noted in eight women as exophthalmic goitre along with palpitations and thyrotoxic symptoms by CH Parry in 1786 but this was only published post-humously much later by his son [2, 3]. A probably underestimated prevalence rate of 1.2% to 9.9% has been reported for autoimmune thyroid disorders in Africa [4, 5]. In South Africa, while thyrotoxicosis (TT) comprised about 66% of all thyroid disease in the country, GD comprised 34% of all thyroid diseases [4]. In Johannesburg, a hospital-based average incidence rate of 8.75 per 100 000 people in women and 0.7 per 100 000 in men was reported for GD in the black population. GD was the most common cause (88%) of thyroid disease with a peak age of incidence between 34 to 54 years. In patients older than 65 years however, toxic multinodular goitre (TMG) prevailed as the most common cause of TT [6]. Typically hyperthyroidism has more often been associated with the female gender. There is a large female predilection (male: female sex ratio up to 1:10), with little documentation in the literature about wholly male hyperthyroid populations [7-9]. A male incidence of 0.00007 per 100, 000 has been reported for South African men while the women have a relatively higher rate of 0.009 [6]. GD is the most common type of primary hyperthyroidism (PH). It is an autoimmune hyperthyroid disease caused by stimulating antibodies against the thyroid stimulating hormone (TSH) receptor which overrule normal control of thyroid hormone production, resulting in hyperthyroidism. These antibodies cause abnormal stimulation of the TSH receptor, resulting in hyperthyroidism [8, 10,11].

Several risk factors have been ascribed to the development of GD including genetic predisposition, pregnancy, stress, and infection [12-14]. GD is diagnosed by the presence of diffuse toxic goiter, biochemical evidence of primary hyperthyroidism, as well as TSH receptor stimulating antibodies [15,16]. The three main methods of managing GD are surgically (thyroidectomy), radioactive iodine-131 (RAI) therapy, and antithyroid medication (ATD) [17]. RAI gradually destroys thyroid follicles by producing a radiation thyroiditis. Common indications for the use of RAI in GD are ATD failure, and recurrent goitre. RAI therapy (RAIT) for hyperthyroidism has been performed for over 80 years, and continues to gain increased acceptance by managing clinicians [18,19]. RAIT is non-invasive, physiologically suitable, and usually does not require hospital admission for the quantity required to treat hyperthyroidism. Non-compliance is not an issue since RAIT is administered directly by the Nuclear Medicine (NM) physician. Side effects are minimal at doses for primary hyperthyroidism. Moreover, due to the tracer quantities of iodine present in the capsule, RAI remains suitable even for patients known with iodine allergies [20]. Although there is no perfect cure for PH yet; the ideal therapy has been described as one which should render patients euthyroid, avoid recurrence of hyperthyroidism, prevent hypothyroidism and de *novo* or recurrent thyroid-associated ophthalmopathy (TAO) [21]. Although hypothyroidism is usually a long-term outcome it may be attained earlier as clinicians give higher quantities of RAI. TAO has been described as worsening after RAI, especially in patients who smoke. This may be mitigated with the use of prophylactic steroids; however Bartalena et al warn that smoking increases the risk of TAO worsening even despite the use of prophylactic steroids [22-24]. There is no documented evidence of differences between male and female genders in response to treatment of PH with RAI, although operational evidence suggests that hyperthyroidism in males is less amenable to RAIT than females. Studies involving wholly male subjects with GD are quite rare, with little evidence in the literature in support of this observation. This study therefore proposed to evaluate male hyperthyroid patients with GD treated at our facility, for factors affecting outcome of RAIT.

## Methods

This is a retrospective analysis of records of hyperthyroid patients who were treated with RAI over a 19-year period at a university teaching hospital, in the Western Cape of South Africa. Patients were typically referred for RAIT from other clinicians. RAIT was preceded in all patients by a Tc-99m pertechnetate thyroid scan to confirm diagnosis of PH, exclude differential diagnoses, estimate uptake of the radiotracer (in lieu of the radioiodine uptake test) and to help plan RAIT. The thyroid scans were performed using a pinhole collimator. RAI doses had been determined empirically, with patients receiving relatively higher doses if older, if diagnosed with toxic multinodular goitre (TMG), or having higher uptake of Tc-99m pertechnetate (PU). For instance, relatively higher doses were administered to patients with TMG than GD, and to older patients than to younger patients. For the purpose of this study, cure was defined as hypothyroidism or euthyroidism at 3 months after receiving RAI. The three-month period was selected based on the pattern of patient return to referring clinician soon after RAIT and thus only a limited number of patients had long enough follow-up. Data analysis was performed with SPSS 21.0 [25]. Analysis of variance (ANOVA) was used to assess the relationship between continuous and categorical data: treatment outcome and patient parameters such as age, Tc-99m pertechnetate uptake, quantity of RAI treatment. Patients' age was stratified in as ≤ 20 years, 21-34 years, 35 to 50 years, and older than 50 years. For categorical analysis, the ages of the patients were further reclassified into those aged 50 years or younger, and those older than 50 years, based on the peak age incidence of 50 years for PH. Estimated thyroid uptake of PU was also grouped as ≤ 4% which is the normal range for patients at our institution, and also as 5-10%, 11-19% and > 20%. The quantity of RAI treatment was also classified as < 250 MBq, 250-399 MBq, and ≥ 400 MBq. Patient ethnicity was classified as Caucasian, mixed origin, Indian, and African; and later reclassified into Caucasian and non-Caucasian for the purpose of categorical data analysis. Comparison of categorical data was performed using contingency tables with the chi-square test. Mean levels of thyroid hormones at presentation and at three-month follow-up were compared using the paired student's t-test. The level of statistical significance was set at 0.05.

## Results

Records of three hundred and sixty-five (365) male patients with primary hyperthyroidism (PH) were available and reviewed. Two hundred and sixty-six (266, 86.4%) had GD. About half (203, 76.3%) had at least three months' follow-up and were considered for statistical analysis. Baseline characteristics of male patients treated for GD are shown in Table 1, and the age distribution of the patients in Table 2. In Table 3, treatment related data are compared for the entire group, the patients who received a single dose of RAI and the patients in this group who were cured after this single dose. Table 4 compares thyroid function tests at baseline and after treatment for the three different groups and shows marked differences in follow-up results mainly favouring cure post-RAIT. The age of presentation for GD was highest in the third to fifth decades of life (126 patients, respectively). Records of patients' pulse rates at presentation were available for 138 patients (68%); we observed tachycardia in 49 (35.5%) of these patients. We further observed that more patients presented without a history of prior ATD use (116, 57.1% versus 87, 42.9%). Most patients (88.2%) had received a single dose of RAI while 20 (9.9%) had two, three had three (1.5%) and one had four doses of RAI (0.5%). Patients of mixed ethnicity comprised the largest proportion (124, 61.1%), followed by Caucasians (61, 30%) then Africans (11, 5.4%) and one Indian patient (0.5%). Ethnicity was not recorded for 6 patients. Following RAIT, one Indian, nine Africans (9.3%), 46 Caucasians (23.4%) and 101 patients (51.3%) of mixed ethnicity were cured. The overall cure rate was 76.4% for these male patients. Cure was observed as euthyroidism in 31 patients (15.3%) and hypothyroidism in 129 (63.5%). Although there were more patients aged 50 years old and below who were cured at three months than older patients (78.9% versus 78.6%) this was not statistically significant (chi square = 0.004, p = 0.95). As with other parameters such as age, thyroid uptake, severity of hyperthyroidism, previous ATD usage, administered quantity of RAI; neither ethnicity (chi square = 1.21, p = 0.75) nor patients' pulse at presentation (chi square = 1.35, p = 0.25) were significant in influencing outcome of RAIT.

**Table 1:** Age at presentation of male hyperthyroid patients treated with RAIT^a^

Age group (years)	Number of patients (frequency (%))	Cumulative total (n)
< 20	7 (3.4)	
21-34	57 (28.1)	64
35-50	69 (34)	133
> 50	70 (34.5)	203

RAIT^a^ = radioactive iodine therapy

**Table 2:** Baseline characteristics of male patients with GD^a^

	AGE	PULSE	PTU^b^ (%)	T4^c^	T3^d^	TSH^e^	RAIT^f^	
Mean	43.58	93.79	16.67	70.09	25.79	0.06	329.23	
Median	44.25	90	14.20	67.10	26.90	0.04	314.00	
Mode	35	80	8	105	31	0	300	
SD^g^	13.23	19.79	10.72	31.06	10.21	0.180	69.15	
Minimum	16	56	1	12	4	0	216	
Maximum	73	160	63	155	44	2	487	

GD^a^ = Graves’ disease

PTU^b^ = pertechnetate thyroid uptake

FT4^c^ = free thyroxine

FT3^d^ = free trioiodothyronine

TSH^e^ = thyroid stimulating hormone

RAIT^f^ = radioactive iodine therapy

SD^g^ = standard deviation

**Table 3:** comparison of baseline parameters of male GD^a^patients, those who received single therapy and those cured after single therapy

Parameter	All GD^a^patients (n = 203)	GD patients administered single dose RAIT^b^ (n = 179)	GD patients cured after single RAIT^b^ (n =152)
**Age at presentation**	43.58±13.23	43.99±13.25	44.1±13.25
**Pulse at presentation (mean ± SD^c^)**	93.79 ± 19.79	92.68 ± 18.76	92.79 ± 19.70
**PTU**^**d**^	16.67±10.72	15.80±9.97	15.6±9.57
**Antithyroid pre-treatment**			
Yes	87 (42.86)	75 (41.90)	63 (41.45)
No	116 (57.14)	104 (58.10)	89 (58.55)
**Number of therapies**			
Single-therapy	179 (88.18)	179 (100)	152 (100)
Multiple-therapy	24 (11.82)	0	0
**First RAIT^b^(mean ± SD^c^)**	329.23±69.15	328.10±69.17	326.98±68.46
**Category of RAIT^b^administered**			
<250 MBq	25 (12.32)	23 (12.85)	18 (11.84)
250-399 MBq	146 (71.92)	129 (72.07)	111 (73.03)
≥400 MBq	32 (15.76)	27 (15.08)	23 (15.13)
Cure			
Yes (euthyroid)		25 (13.97)	25 (16.45)
Yes (hypothyroid)		127 (70.95)	127 (83.55)
No		27 (15.08)	

GD^a^=Graves’ disease

RAIT^b^ = radioiodine therapy

SD^c^ = standard deviation

PTU^d^ = estimated Tc-99m pertechnetate uptake

**Table 4:** Comparison of baseline and post-treatment thyroid function tests among all male GD^a^ patients, those who received single therapy and those cured after single therapy

	TSH^b^		FT4^c^		FT3^d^	
Variable median (range)	Baseline	3 months	3 months	3 months	Baseline	3 months
**All GD^a^**	0.04	11.43	2.9	5.8	26.9	2.9
	(0-2)	(0-150)	(0-105)	(0-130)	(4-44)	(0-105)
**GD with single treatment RAI^e^**	0.04	20.02	2.7	5.25	26.6	2.7
	(0-2)	(0-150)	(0-105)	(0-81)	(4-44)	(0-105)
**GD with single treatment RAI^e^and cured**	0.04	26.32	2.40	4.6	27.35	2.40
	(0-2)	(0-150)	(0-10)	(0-26)	(4-44)	(0-10)

GD^a^ = Graves’ disease

TSH^b^ = thyroid stimulating hormone

FT4^c^ = free thyroxine

FT3^d^ = free triiodothyronine

RAI^e^ = radioactive iodine

## Discussion

As GD is more commonly encountered in women, there are few studies that have studied GD in male patients. One such study by Blahd and Hays was performed on 241 wholly male patients of heterogeneous ethnic origin with GD [26]. These men were aged 21 to 78 years, with an average age of 44 years. Eighty-five percent of treated patients became euthyroid after a single dose of RAI ranging from 55-770 MBq. This was a long-term study with follow-up for up to 17 months after RAIT. It was observed that African patients required more treatment sessions with RAI than Caucasians (74% vs. 45%), which reflected more severe GD in the former. The quantity of administered RAI did not affect the outcome of RAIT. In another mixed population of 464 male and female African patients (female-to-male ratio of 8.5:1) GD comprised 88% of the patients [6]. The authors observed that male hyperthyroid African patients tended to present at an older age than their female counterparts by five years. Patients with GD had an average age of 39.9 ± 11.3 years in women and 44.7 ± 14.1 in men. The male: female sex ratio was approximately 1:8. In this present study, GD comprised 73% of all male hyperthyroid patients referred for RAIT, which was in keeping with reputed incidence of 50-88% [6, 8]. The average age at presentation of these patients was 43 years (with a range of 16-73 years), also consistent with previously reported age incidence of GD [6].

Within three months of being treated with a range of 216 to 487 MBq of RAI (average of 329 MBq), 76% of these patients had been rendered euthyroid or hypothyroid. Considering that this was a shorter follow-up period than observed in the earlier study by Blahd and Hays, this result would seem to indicate that higher cure rates would have been observed with longer follow-up. The more hyperthyroid presentation of our patients could be explained by the fact that they had all been referred for RAIT, one of the indications for which was hyperthyroidism recalcitrant to ATDs. This contrasted a West African study in which the patients presented largely with ATD-induced euthyroidism/hypothyroidism which also reflected in patients' body-mass index (BMI) [27]. It has also been observed that male patients with GD tended to have a more severe presentation than females [28]. RAIT for hyperthyroidism began in the early 1940s and has been described as an initial application of nuclear medicine [29].

After incorporation into the thyroid, emitted beta radiation causes gradual necrosis of thyroid tissue [30]. Despite its advantages, onset of cure is slower compared to thyroidectomy and thus patient follow-up is necessary to ascertain the time of cure [21]. Follow-up after RAIT requires adequate time to assess efficacy of therapy. In this study, follow-up was limited to 3 months due to the patient attrition. As has been mentioned previously, the practice of patients returning to their referring/managing physicians for further management, and local transportation challenges contributed significantly to attrition. Knowledge of outcome is necessary to determine the benefits of RAIT. Patients who remain hyperthyroid after treatment with RAI at this time (three months) will be closely monitored as they may require re-treatment with RAI. The decision to repeat RAIT is made with persistent hyperthyroidism up to six months to one year post-RAIT; most patients would have been cured by six months [31].

The quest for prediction of outcome of RAIT is a perpetual one. Certain factors have been alluded to as influencing outcome of therapy. These are patients' age and gender, pretherapy with antithyroid medication, thyroid size as well as thyroid-associated ophthalmopathy [27, 28, 32]. Transient hypothyroidism has been observed in a small proportion (11-17%) of hyperthyroid patients within a few months of RAIT [33-35]. Thyroid hormone levels initially decrease and may then spontaneously resolve to euthyroidism or rebound to hyperthyroidism. However, basal TSH levels from 45 mU/L and above after RAIT have been described as excluding patients from this phenomenon with a specificity of 100% [33]. This may serve as a guide for managing clinicians. The cure rate of 76% in this study is similar to that found in a study of 605 male and female patients with GD in the United States [32]. A success rate of 85% was also found in 241 male patients with GD [26]. In another subset of 389 GD patients (from a mixed population of 555 patients with GD, TMG and toxic adenoma), 90% required only one therapy session with RAIT to be cured [36]. A similar result of 90% was obtained in a questionnaire survey of patients with GD and toxic nodular goitre [37]. A smaller study from West Africa has shown a rate of 77% [38]. Thus, there is documented evidence of high success rate following a single dose of RAI for GD. In the previous study on wholly male patients, patients aged 21-78 years received empirical doses as little as 1.5-20.8 mCi as first dose of RAI. The study also affirmed that the dose of RAIT delivered had little effect on therapeutic outcome. The most distinctive feature between patients who were cured with a single dose of RAI and those requiring multiple doses was ethnic origin; 74% of patients of African descent needed further therapy, compared to 41% of those other ethnicities. African patients also displayed greater weight loss, protein-bound iodine, RAI uptake, and required a significantly longer time to be cured (euthyroidism and hypothyroidism) [26]. However, in our study, ethnicity was not influential in determining outcome of RAIT.

## Conclusion

Graves' disease in men remains uncommon. In this cohort of hyperthyroid patients, the factors which have been evaluated as affecting outcome of RAIT were unimportant. Despite the mainly hyperthyroid presentation of the patients, RAIT was so effective that the main type of cure after therapy was hypothyroidism.

### What is known about this topic

- Radioactive iodine treatment is effective in the management of primary hyperthyroidism including Graves' disease.

### What this study adds

- A wholly male heterogeneous South African study adds further evidence that radioactive iodine-131 therapy is effective in male patients with Graves' disease;
- In this South African population, African patients tended to require multi-treatment to be cured of hyperthyroidism;
- Radioactive iodine-131 therapy still effective despite mainly hyperthyroid presentation of patients.

## Competing interests

The authors declare no competing interests.
